# Rapid Separation of Human Hemoglobin on a Large Scale From Non-clarified Bacterial Cell Homogenates Using Molecularly Imprinted Composite Cryogels

**DOI:** 10.3389/fbioe.2021.671229

**Published:** 2021-10-01

**Authors:** Solmaz Hajizadeh, Karin Kettisen, Leif Bülow, Lei Ye

**Affiliations:** Division of Pure and Applied Biochemistry, Department of Chemistry, Lund University, Lund, Sweden

**Keywords:** hemoglobin, purification, composite cryogel, molecularly imprinted polymer, scaling up

## Abstract

The production of a macroporous hydrogel column, known as cryogel, has been scaled up (up to 150 mL) in this work for the purification of human hemoglobin from non-clarified bacterial homogenates. Composite cryogels were synthesized in the presence of adult hemoglobin (HbA) to form a molecularly imprinted polymer (MIP)network where the affinity sites for the targeted molecule were placed directly on an acrylamide cryogel by protein imprinting during the cryogelation. The MIP composite cryogel column was first evaluated in a well-defined protein mixture. It showed high selectivity toward HbA in spite of the presence of serum albumin. Also, when examined in complex non-clarified *E. coli* cell homogenates, the column showed excellent chromatographic behavior. The binding capacity of a 50 mL column was thus found to be 0.88 and 1.2 mg/g, from a protein mixture and non-clarified cell homogenate suspension, respectively. The recovery and purification of the 50 mL column for separation of HbA from cell suspension were evaluated to be 79 and 58%, respectively. The MIP affinity cryogel also displayed binding and selectivity toward fetal Hb (HbF) under the same operational conditions.

## Introduction

Purification of recombinant proteins from bacterial or yeast extracts often represents a major bottleneck in a biotechnology driven production system. Particularly, the purification of intracellular proteins is far from trivial and multiple chromatographic, or precipitation steps are often needed. The number and selection of steps to achieve a pure product are dependent on the size, charge, solubility and other properties of the target protein. However, the first step in purifying intracellular proteins is the preparation of a crude extract. This extract will contain a complex mixture of all the proteins from the host cells, nucleic acids, cellular metabolites and lipids. Crude cellular protein extracts are prepared by the removal of cellular debris generated by cell lysis, which is achieved using chemicals, enzymes, sonication or a French Press. The obtained debris is removed by centrifugation or filtration, and the supernatant is recovered. This step is labor intensive and requires handling of large volumes. When the entire protein purification scheme is evaluated, the clarification of crude cellular extracts often represents the most time-consuming step in the whole process. In the present study, we have examined the possibility of omitting centrifugation and directly apply the crude extracts after cell lysis on a chromatographic column. A conventional matrix would rapidly clog when handling such complex mixtures. By exploring cryogels with exceptionally large pore sizes, we could develop a material suitable for managing very crude starting protein solutions.

The formation of such macroporous hydrogels, known as cryogels, at sub-zero temperature was reported ∼50 years ago (Lozinsky, 2002). The cryotropic gelation process can be summarized as following (Lozinsky et al., 1984; Carvalho et al., 2014; Sun et al., 2020), (a) the reaction mixture is frozen below the solvent crystallization point, which contains unfrozen microspheres and crystals of the frozen solvent; (b) the concentration of the precursors increases dramatically in the microspheres and begins the polymerization; (c) the frozen sections act as a pore-forming agent and form macropores after being melted; (d) the formed polymeric network is interconnected since the solvent crystals grow until they meet the facet of another crystal. The large interconnected channels in the matrix allow passing particulate-containing fluids such as blood (Derazshamshir et al., 2010), wastewater (Önnby, 2016), cell suspensions (Zhou et al., 2015; Hajizadeh et al., 2018) with low backpressure. This unique property has attracted attentions in biotechnology (Saylan and Denizli, 2019; Baimenov et al., 2020) and biomedicine (Jagur-Grodzinski, 2010; Mattiasson et al., 2010; Henderson et al., 2013; Kumar, 2016b).

The main challenge is scaling up these macroporous hydrogels, which can be later applied for industrial use. The main factor has a correct freezing pattern. The formation of the first ice nucleus in the polymerization solution to growing ice crystals in the whole sample should occur rapidly (Plieva et al., 2004a,b). By increasing the diameter of the column, freezing time will take longer due to the presence of a temperature gradient inside the polymerization mixture. In this case, the part of the solution closest to the cooling source has a lower temperature than the center of the mixture. While the outer layers are frozen, the polymerization starts and forms a hydrogel in the center of the mixture (Lozinsky, 2014). This affects the final morphology and the mechanical stability of the material (Plieva et al., 2004a). Recently, Savina et al. (2016) reported preparing a large volume of cryogel (up to 250 mL) by adding the initiator into the monomer and ice crystal suspension. The advantage of this approach is the possibility of scaling up the macroporous hydrogel to the desired volume. However, the disadvantage of the method is that when the solution is partially frozen before the initiator’s addition, the initiator may be unevenly distributed, which can cause diverse levels of mechanical stability in different parts of the gel (Savina et al., 2016). This phenomenon may affect the column’s regeneration and can be noticed even more when forming a composite cryogel using particle suspension.

Using molecularly imprinted polymer (MIP) composite cryogel for recognition and removal of protein has been reported in the literature (Andaç et al., 2012; Fan et al., 2018; Venkataraman et al., 2020). The MIP technique involves the polymerization of functional monomers around a target molecule. When the reaction was completed, the target is removed and leaves behind an artificial site with specific interaction toward the adsorbate (Pan et al., 2021). Forming MIP composite cryogel directly on the surface of the network was reported by our group earlier (Hajizadeh et al., 2018).

In this study, we have scaled up cryogel composite column by 300-fold for purification of HbA from both a well-defined protein mixture and a crude lysed *E. coli* cell suspension in this work. Based on the authors’ knowledge, it has been the first time that a scaled-up composite cryogel column has been utilized to separate a biomolecule from non-clarified cell homogenate suspension. The binding capacity of the columns, as well as selectivity toward the target protein, were examined in this study. The chromatographic columns could be regenerated and reused at least five times. Forming a robust, mechanical stable column that can shorten the downstream process and produce reliable data are some of the advantages of using a cryogel network at the industrial scale.

## Materials and Methods

### Materials

Acrylamide (Am), N, N, N’,N’-tetramethylethylenediamine (TEMED), N, N’-Methylenebis (acrylamide) (MBAm), human adult hemoglobin (HbA, lyophilized powder), acetic acid, bovine serum albumin (BSA), sodium phosphate, sodium carbonate, ammonium per sulfate (APS), sodium hydrogen carbonate, sodium hydrogen phosphate and sodium dodecyl sulfate (SDS) were purchased from Sigma-Aldrich, Sweden. *E. coli* cells were cultured in-house following an optimized procedure of a previously published protocol for preparation of recombinant HbF (Reeder et al., 2008). Briefly, *E. coli* BL21 (DE3) cells, containing a pETDuet-1 plasmid from Novagen for co-expression of genes coding for the alpha and gamma chain of HbF (Ratanasopa et al., 2016), were grown in 300 ml Terrific Broth in 1 l Erlenmeyer flasks. The cells were harvested by centrifugation and washed with 0.1 M phosphate buffer pH 6.0 prior to aliquoting and freezing in liquid nitrogen for storage before experiments. Per experiment, 1 g cells were thawed and resuspended in the aforementioned phosphate buffer before disintegration by sonication. After bubbling the sonicated suspension with CO gas to stabilize the Hb proteins, the concentration of HbF was calculated with a three-point drop baseline correction of the Soret peak at 419 nm. The concentration of HbF was adjusted to 0.5 mg/ml by diluting with buffer before use.

### Preparation of Cryogel

Acrylamide cryogel has been prepared using radical polymerization (Hajizadeh et al., 2018) with a total monomer concentration of 6% and a 6:1 molar ratio between Am and MBAm. TEMED was added to the mixture, forming 2% of the total monomer volume. Forty-nine milliliter aliquots of the solution were transferred into a plastic syringe (i.d., 3 cm) and degassed by purging nitrogen, while the solution was cooled down on an ice bath for a minimum of 20 min. One milliliter of ammonium per sulfate (APS) solution (2% of the total monomer volume) used as an initiator was degassed under nitrogen gas and quickly mixed with the monomer solution in the final step. The mixture was frozen and kept at −12°C in a liquid cryostat overnight. The gels were defrosted at room temperature and thoroughly washed with water to remove all un-reacted monomers. The macroporous hydrogel was noted as “NIP Am-CG” and used as a control.

The imprinted cryogel, named “MIP Am-CG,” was prepared under the same conditions except that HbA was added to the monomer solution (the molar ratio of Am:HbA was 1:0.5). After polymerization, the imprinted cryogel was washed with 50 mL of acetic acid solution (10% v/v) containing SDS (5% w/w) to remove the template. This concentration of acetic acid and surfactant was kept constant in all experiments for template removal and column regeneration. The gels were then washed with water until the pH became neutral and dried in an oven at 60°C.

### Characterization of Cryogel

Equation 1 was used to calculate the polymerization yield (Y)of the cryogel:


(1) $$\text{Y}\left(\%\right)=\frac{{\text{W}}_{1}}{{\text{W}}_{0}\times 100}$$

where W_0_ and W_1_ are the theoretical mass and the mass of the dried cryogel, respectively. Dried cryogel was weighed and soaked in distilled water for 1 h at room temperature to determine the swelling degree of the gels. Then, excess surface water was removed using a filter paper, and the swollen cryogel was weighed. The swelling degree (S_w_) was calculated using Equation 2:


(2) $$S_{w}=\frac{{\text{W}}_{2}-{\text{W}}_{1}}{{\text{W}}_{1}}$$

whereW_1_ and W_2_ are the weight of the dried and swollen cryogel, respectively. The structure of the cryogels was studied by scanning electron microscopy (SEM) using a Hitachi SU3500 (Hitachi, Japan) at 10 kV. The samples were taken from the center of the cryogel’s middle section and coated with a thin (15 nm) layer of gold prior to SEM imaging. Fourier transform infrared (FT-IR) spectroscopy (Nicolet iS5, Thermo-Fisher Scientific Inc., Waltham, MA, United States) was used to study the chemical changes after each synthetic step, with a resolution of 4 cm^–1^ and 16 scans.

### Adsorption and Elution in a Chromatographic Setting

#### 50 mL Column

Hemoglobin adsorption/elution experiments were performed in a chromatographic setting. Aplastic column (i.d., 3 cm) fitted with a flow adaptor at its end was used. Solution/suspension mixtures were pumped out of the column using a peristaltic pump. The flow rate was set at 2 mL/min throughout all experiments unless stated otherwise. Void volume was calculated to be equal for all the setups. Hence its impact was disregarded in this study. The absorbance of the outlet stream was monitored continuously with a spectrophotometer at 405 nm wavelength. The selectivity of the MIP affinity chromatography column was evaluated in the presence of a protein mixture (1 mg/mL HbA and 3 mg/mL BSA). The gels were washed with 30 mL of a running buffer consisting of phosphate buffer (0.1 M, pH 6.0) until the baseline on the UV spectrometer became stable. Thirty milliliter of a protein solution was added to the column to study the breakthrough curve. Sixty mL of the running buffer was applied to remove any non-specific bound proteins from the column. Hemoglobin was eluted from the column using 40 mL of carbonate buffer (0.1 M, pH 9.0). The affinity column was regenerated by passing acetic acid and SDS solutions through the gels. Fractions from each step were collected for SDS-PAGE analysis.

The purification of the target molecule was evaluated under more realistic conditions by suspending *E. coli* cells (1.5 g) in 30 mL of the running buffer. The cells were disintegrated using sonication pulses. The sonication of cells was performed for 2 min while the container was kept in an ice bath with 2 s pulses followed by 3 s of interruption. The process was started at 50% amplitude and gradually increased to 95% during the 2 min of operation. The obtained cell suspension was diluted with the same buffer to 60 mL and named as non-clarified cell homogenate, which was then spiked with 60 mg of HbA. The same chromatographic process described above was followed to purify the HbA from the suspension, and the elution fraction was collected for SDS-PAGE analysis.

For purification of HbF directly from the non-clarified cell homogenate, a suspension of 0.5 mg/mL cell suspension in a running buffer was prepared. The cell mixture underwent no further treatment, and it was used directly as a loading sample (20 mL). The column was washed with 30 mL of the running buffer before the elution step using 20 mL of carbonate buffer (0.1 M, pH 9.0).

Samples were electrophoresed on a 4–20% polyacrylamide gradient Tris-glycine gel for 90 min at 100 V using a Bio-Rad electrophoresis cell. The gel was stained in Coomassie blue for 1 h, then destained for 24 h in a solution of methanol and acetic acid. Image Lab software (Version 6.1, Bio-Rad) was used for processing the protein bands on the SDS-PAGEs.

### 150 mL Column

The volume of the column was increased threefold by stacking 50 mL cryogel columns inside a Bio-rad column (i.d., 3 cm) to improve the binding capacity. The column was then washed with 60 mL of the running buffer. Protein solution (50 mL) was passed through the column to study the breakthrough curve. The column was washed with the phosphate buffer (0.1 M, pH 6.0) until it reached the baseline and removed any non-specifically bound proteins. Hemoglobin was eluted from the column using 50 mL of carbonate buffer (0.1 M, pH 9.0).

For the separation of HbA from particulate-containing fluid, 2 g of *E. coli* cells were broken down by sonication in 100 mL of phosphate buffer (0.1 M, pH 6). The non-clarified crude suspension (50 mL) was spiked with 50 mg HbA. For this experiment, the flow rate of the pump was adjusted to 1 mL/min. Fifty mL of the spiked mixture was loaded onto the column. Phosphate buffer (100 mL, 0.1 M, pH 6) was used to remove the non-specific binding impurities, and 75 mL carbonate buffer (0.1 M, pH 9) was applied for the elution of bound HbA.

## Results and Discussion

### Characterization of the Cryogel

**FIGURE 1:**
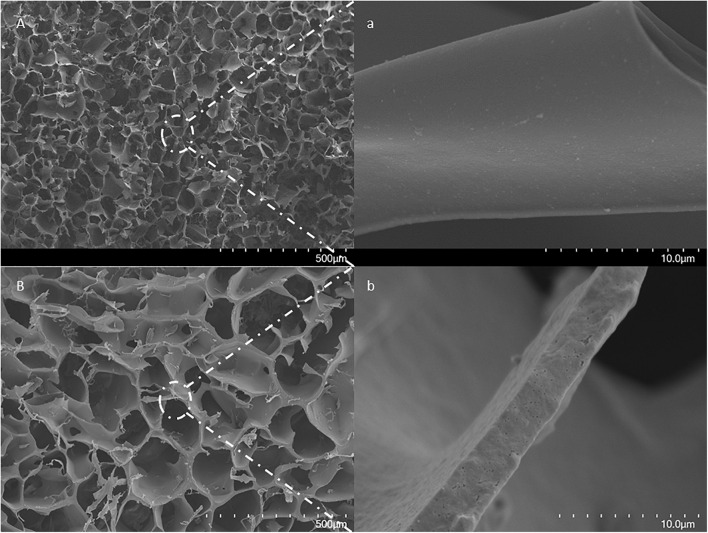
SEM images of a cross-section of a formed MIP Am-CG by **(A)** traditional approach for small-volume; **(B)** modified method for larger volume. The scale bars show 500 μm and 10 μm for low and high magnifications, respectively.

The advantages of the large and interconnected channels in cryogels have been studied and reported elsewhere (Plieva et al., 2011; Carvalho et al., 2014; Baimenov et al., 2020; Sun et al., 2020). This material has a unique construction for separation purposes, especially when dealing with particulate-containing fluids such as cell homogenates and wastewater (Hajizadeh et al., 2014, 2018; Önnby, 2016; Srivastava et al., 2016). To avoid diverse mechanical stability in the columns (Savina et al., 2016), in this work, the monomer solutions were, therefore, cooled down to below zero, and the APS solution was added before the formation of any visible ice crystals. The scanning electron microscopy (SEM) image of the cryogel is shown in Figure 1. The sample was selected from the central area of the middle section of the column. Small nanometer-sized holes can be seen at high magnification on the surface of the gel. These nanopores may result from the pre-cooling effect and the formation of the ice nucleus prior to polymerization.

In contrast, in the traditional method of cryogel formation, the wall surfaces are smooth and continuous. Thus, the alternation on cryogel preparation shown in this work may still impact the structure of the cryogel without dramatically jeopardizing the mechanical stability of the column. The effect of the introduction of HbA to form MIP composite cryogel can be seen in the FT-IR spectra (Supplementary Figure 1). Most of the HbA bands and the iron groups in the protein (ferric form) are overlapped with NIP Am-CG. However, one of the ferric oxide bands, around 928 cm^–1^ (National Institute of Standards and Technology (NIST), 2018), can distinguish the difference between the imprinting and non-imprinting column (Supplementary Figure 1).

Table 1 lists the physical characteristics of the control NIP and MIP Am-CGs. The flow rates of both matrices were assessed by passing water through the column. Compared with the 0.5 mL column (Hajizadeh et al., 2018), only a 5% decrease in water passage through the NIP Am-CG after scaling up the column. However, the flow rate was reduced by 29% when the volume was increased from 0.5 to 50 mL for the composite cryogel MIP Am-CG. This may be explained by the presence of the protein in the initial solution, which can impact the polymerization reaction and the final configuration of the channels. The polymerization yield and swelling of both MIP and NIP gels are similar to other types of cryogels that have been reported elsewhere (Carvalho et al., 2014; Kumar, 2016a; Farías et al., 2020).

**TABLE 1:** Synthesis of cryogels and characterization of physical properties.

NIP Am-CG	10:0	90.0 ± 1	6.02 ± 0.1	3.5 ± 0.05
**MIP Am-CG**	10:1	87.6 ± 3	7.71 ± 0.1	2.7 ± 0.07

### Chromatographic System (50 mL Gel)

The concentration of HbA was monitored using a spectrophotometer at 405 nm. The molar extinction coefficient was calculated to be (ε_405_) 147 mM^–1^ cm^–1^. The adsorption isotherm for both gels has been reported before (Hajizadeh et al., 2018). In this study, we focused on scaling up the system for application under realistic operation conditions. Supplementary Figure 2 shows the NIP Am-CG column during the adsorption and elution procedure of HbA from the protein mixture. As is shown in the figure, the color of the NIP Am-CG changed from white to light brown after loading the protein mixture. Supplementary Figures 2B,C,E, were taken from different angles of the column after further purification steps. In all three images, the uniform distribution of the color (protein mixture) indicates that the entire surface area of the column contributes to the adsorption. It should be stressed that the heme group here is mainly in a ferric state and thus has a brown color with the highest absorption peak at 405 nm.

**FIGURE 2:**
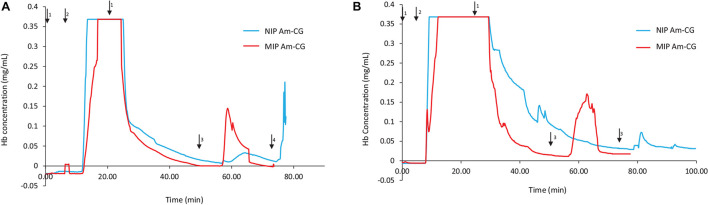
Chromatograms of adsorption and elution of HbA from **(A)** protein mixture; **(B)** spiked non-clarified cell homogenate. Chromatograms of NIP Am-CG and MIP Am-CG were shown by blue and red lines, respectively. The arrows indicate the introducing of different solutions during the process: (1) 0.1 M phosphate buffer pH 6, (2) loading sample in the running buffer, (3) 0.1 M carbonate buffer pH 9, (4) Acetic acid (10% v/v) with SDS (5% w/w). In **(B)**, the peaks around 50 min on the NIP Am-CG were caused by an air bubble inside the flow cell.

Figure 2 shows the chromatograms for both NIP and MIP Am-CGs. The concentration of the HbA in the protein mixture was above the measuring limit of the spectrophotometer and the breakthrough curve thus became flat at the top of the peak. During the loading step, the HbA protein came out of the imprinted column with some delay compared to the non-imprinted column (Figure 2A). Due to the higher selectivity of the MIP Am-CG column, more HbA proteins adsorbed during the loading process, and less was found in the flow-through stream. The higher elution peak of HbA confirmed this assumption in the MIP Am-CG compared to the non-imprinted cryogel (Figure 2A). The appearance of a peak after 80 min on the NIP Am-CG chromatogram is related to the regeneration of the column by acetic acid and SDS. This peak was a consequence of the refractive index inside the flow cell when two solutions with different viscosities were mixed (the carbonate buffer and the acetic acid solution containing SDS). The regeneration step with the acid and surfactant is included in all experiments but is only shown in Figure 2.

The same adsorption/elution practice on the 50 mL columns was carried out with non-clarified cell homogenate spiked with HbA. Figure 2 shows the chromatograms for both types of columns. Compared to Figure 2A, the washing step involving the running buffer to remove non-specific bound proteins was longer for both columns. The NIP Am-CG had to be washed with extra running buffer (20 mL) in order to ensure that the outlet stream from the column reached the baseline and that there were no impurities left on the gel. The polymer structure of the non-imprinted gel may explain the need for this extra step. The imprinted cryogel was designed to be more specific in recognizing HbA proteins. In the MIP Am-CG, HbA protein has the priority to adsorb to the wall’s surface via the imprinting effect of the target protein during the polymerization process (Hajizadeh et al., 2018). This minimizes the chances that other proteins in the suspension have of interacting with the column in the first place. Thus, the step of washing the MIP Am-CG column with the running buffer to remove the other molecules is faster. On the other hand, polyacrylamide is hydrophilic and can interact with proteins, cells, etc. (Carvalho et al., 2014). The non-imprinted cryogel does not have any preference for the target protein (HbA, in this study). Therefore, this protein and other impurities in the non-clarified cell homogenate suspension must compete for adsorption to the cryogel. In this case, the competing host cell proteins adsorbed to the cryogel, possibly via multipoint attachments (Memic et al., 2019), making it more difficult to remove them with the running buffer in the washing step. As expected, the elution peaks from the MIP Am-CG were more extensive than those of the NIP Am-CG (Figure 2B). Based on the breakthrough curves, the amount of the bound HbA protein was calculated from the elution peak by using the OriginPro^TM^ 2019 software (Table 2). The concentration of the bound HbA was higher on the MIP Am-CG from both the protein mixture solution and the non-clarified cell homogenate suspension.

**TABLE 2:** The amount of HbA bound to the column cryogels.

Cryogel type	Column (mL)	Amount of bound HbA (mg/g gel)		Recovery (%)		Purification (%)	
				
		Protein solution	Cell homogenate spiked with HbA	Protein solution	Cell homogenate spiked with HbA	Protein solution	Cell homogenate spiked with HbA
MIP-Am cryogel	50	0.88 ± 0.05	1.2 ± 0.1	68.0 ± 1.30	79.1 ± 2.70	47 ± 2	56 ± 3
NIP-Am cryogel		0.23 ± 0.005	0.45 ± 0.007	34.8 ± 2.26	31.6 ± 2.43	38 ± 3	30 ± 2
MIP-Am cryogel	150	1.27 ± 0.51	1.23 ± 0.76	70.8 ± 2.31	74.3 ± 2.51	40 ± 5	46 ± 3
NIP-Am cryogel		0.92 ± 0.03	0.31 ± 0.06	37.4 ± 1.95	28.1 ± 0.07	15 ± 4	33 ± 1

Compared with our previous study at a smaller scale (Hajizadeh et al., 2018), by increasing the volume of the column by 100-folds, regardless of the flow rate, the entire separation process increased from 25 to 70 min. It should be noted MIP cryogel column at this scale has not been reported elsewhere. Thus, the amount of the bound proteins on the column may not be compared with other reported MIP cryogels for HbA separation (Derazshamshir et al., 2010; Baydemir et al., 2014; Dolak et al., 2020), due to the different operational conditions and the size of the columns.

**FIGURE 3:**
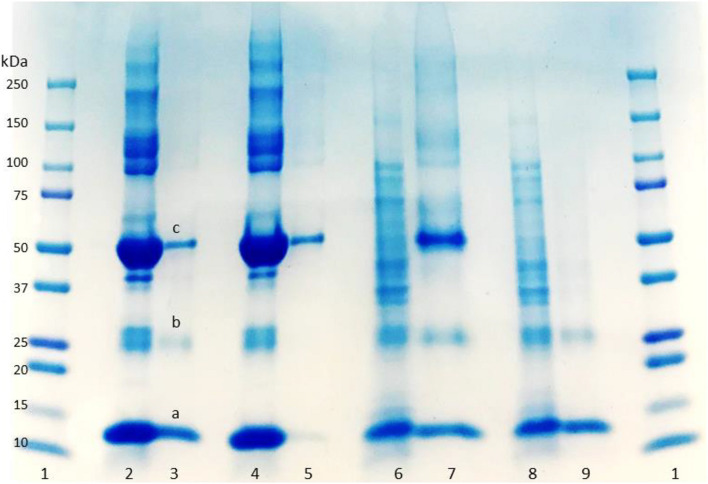
Digital image of SDS-PAGE analysis of protein mixture and spiked non-clarified cell homogenate with HbA. (1) Protein marker; (2) protein mixture loaded on MIP Am-CG; (3) elution fraction from MIP Am-CG; (4) protein mixture loaded on NIP Am-CG; (5) elution fraction from NIP Am-CG; (6) HbA-spiked non-clarified cell homogenates loaded on NIP Am-CG; (7) elution fraction from NIP Am-CG; (8) HbA-spiked non-clarified cell homogenates loaded on MIP Am-CG; (9) elution fraction from MIP Am-CG. The bands marked as a, b, and c correspond to the monomer, dimer and tetramer structures of HbA with a molecular weight of 16, 32, and 65 KDa, respectively.

SDS-PAGE was used to evaluate the purity of the eluted protein from the column. Lanes 2 and 4 in Figure 3 display the BSA and HbA protein solution bands, respectively. BSA has a band around 66 KDa, and HbA appears in three different bands at 65, 32, and 16 KDa, which represent the tetramer, dimer and monomeric forms of the protein, respectively. The other bands in the loading lanes represent other blood proteins. With regards to the purification of the non-clarified cell homogenate suspension, the presence of the impurities alongside the HbA proteins can be seen in the NIP Am-CG column (Figure 3, lane 7). This indicates that plain Am-CG has no specific binding sites for HbA proteins. The eluted fraction from the imprinted column shows only dimer and monomer bands of the HbA proteins at 32 and 16 KDa, respectively (Figure 3, lane 9). No additional bands related to impurities could be detected from this elution on the SDS-PAGE. This quality control of the elution fractions from the 50 mL MIP Am-CG column confirms the selectivity of the column toward HbA adsorption.

The concentration of the eluted protein, the purification factor and recovery percentage of HbA from each column are summarized in Table 2. In comparison with available reports in the literature (Aboul-Enein, 1999; Lu et al., 2004), the purity and recovery percentage of the column might not be impressive at first glance. However, the integration of several steps in the separation process in downstream processing to shorten the time, minimize the cost as well as the potential loss of the product between each step is attractive to large scale applications. In this work, the imprinted cryogel column has recovered about 79% of the protein in a duration of 40 min in the first step of the hemoglobin separation from non-clarified cell homogenate suspension while increasing the purity from 20 to 58%. It should be mentioned that both types of loaded samples (protein solution and spiked non-clarified cell suspension) reported in Table 2 are artificial samples where the concentration of the target protein in both of them is already high from the start.

### Chromatographic System (150 mL Gel)

**FIGURE 4:**
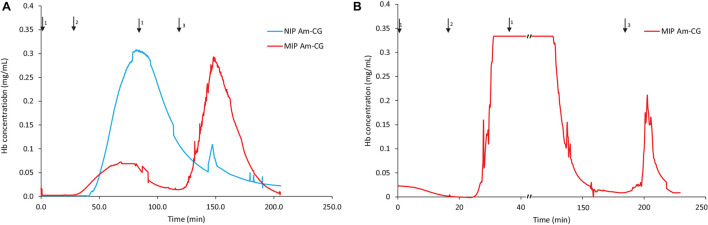
Chromatograms of adsorption and elution of HbA from **(A)** a protein mixture; **(B)** spiked non-clarified cell homogenate. Chromatograms of NIP Am-CG and MIP Am-CG were shown by blue and red lines, respectively. The arrows indicate the introducing of different solutions during the process: (1) 0.1 M phosphate buffer pH 6, (2) loading sample in the running buffer, (3) 0.1 M carbonate buffer pH 9, (4) Acetic acid (10% v/v) with SDS (5% w/w).

The volume of the columns was increased threefold by stacking 50 mL of cryogels inside a Bio-Rad chromatography column (Supplementary Figure 3). The focus was on studying the behavior of the cryogel columns in HbA adsorption/elution procedures from different media. The breakthrough curves for both the protein mixture and the non-clarified cell homogenate suspension spiked with HbA are displayed in Figure 4. Cryogel is a soft and elastic material, and this characterization makes the applications of the gel unique and popular in different fields such as biomedical and tissue engineering (Kumar, 2016b; Memic et al., 2019). In this chromatography setup configuration, due to the negative pressure applied inside the column during the separation process, the bottom gel could be squeezed, having an impact on the total volume of the column and its banding capacity. Thus, the elasticity of the macroporous hydrogel can be considered a disadvantage in this experimental design. The flow rate of the pump was decreased from 2 to 1 mL/min to address this challenge. It should be noted that this is a home-made chromatography setup, and while running the NIP Am-CG, due to the presence of bubbles in the flow-through cell, the breakthrough curve was distorted to be presented. However, each fraction was collected separately for further analysis. The amount of adsorbed HbA in each column based on the chromatograms is summarized in Table 2.

The MIP Am-CG column was washed with 150 mL of running buffer to remove cells, cell debris and other impurities from the column. It was noticed that diluting the loading suspension to reduce its viscosity helps decrease the washing period and minimize the risk of the cryogel collapsing inside the column.

**FIGURE 5:**
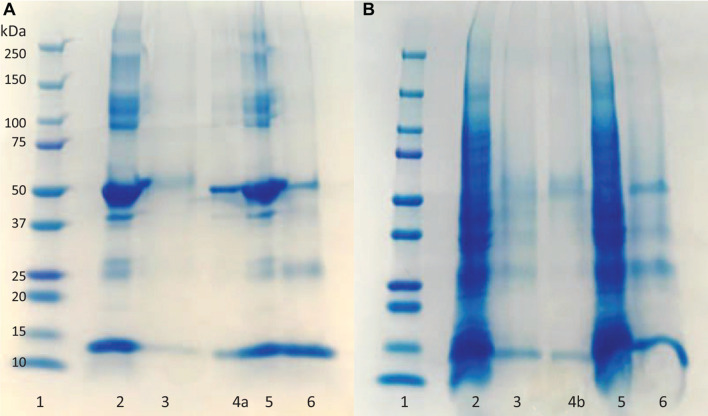
Digital image of SDS-PAGE analysis **(A)** protein mixture; **(B)** spiked cell homogenate with HbA. (1) Protein marker; (2) loaded sample on NIP Am-CG; (3) elution fraction from NIP Am-CG; (5) loaded sample on MIP Am-CG; (6) elution fraction from MIP Am-CG; (4a and 4b) are the diluted fraction of the lanes (6A and 3B), respectively.

The fractions of the loading and elution steps from the column were assessed by SDS-PAGE (Figure 5). Similarly to the 50 mL columns, both types of macroporous hydrogel (NIP and MIP) can separate the HbA under these operating conditions. However, MIP Am-CG has a higher selectivity toward HbA. HbA bands (16, 32, and 68 KDa) are therefore more easily detectable in Figures 5A,B, lane 6 in comparison to the lanes A3 and B3, which belong to NIP Am-CG eluted fractions. Hence, increasing the volume of the column does not affect the purification factor (Table 2).

### Separation of Fetal Hemoglobin (HbF) by MIP Am-CG (50 mL)

**FIGURE 6:**
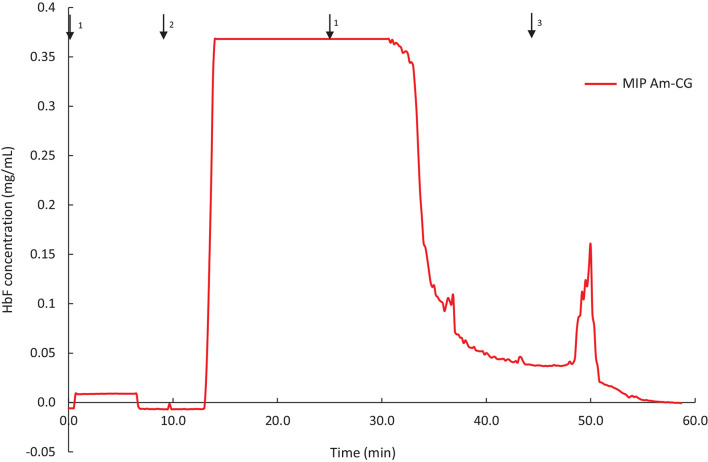
Chromatograms of adsorption and elution of HbF from non-clarified cell homogenate using MIP Am-CG column (50 mL). The arrows indicate the introducing of different solutions during the process: (1) 0.1 M phosphate buffer pH 6, (2) loading sample in the running buffer, (3) 0.1 M carbonate buffer pH 9.

The MIP Am-CG column was prepared in the presence of HbA and has shown selectivity toward the template molecule both in a well-defined protein mixture and a complex cell suspension. To further characterize the chromatographic material, HbF was also used as a test sample for addressing the selectivity potential of the cryogel. HbF and HbA are both tetrameric proteins in their native form. They both contain two α harbors two γtwo *upbeta*chains present in HbA. The column was assessed to separate and HbF from a non-clarified cell homogenate. The breakthrough curve of the column is presented in Figure 6.

The binding capacity of the column for HbF under the described conditions was 0.62 ± 0.04 mg per g of gel. The purification and recovery of the HbF were calculated to be 28 and 68%, respectively. Although the amount of the bound protein is in range with the HbA (Table 2), but the purification and the recovery of the proteins, based on SDS-PAGE analysis, are much lower (Supplementary Figure 4). This lower affinity of the MIP Am-CG column toward HbF can be explained by the imprinting effect of the HbA protein, which was used as a template in the first place. The column, therefore, exhibits a higher sensitivity toward the HbA than HbF proteins. The ability to recognize HbA from HbF using MIP particles has previously been reported by our group (Zhang et al., 2019).

**FIGURE 7:**
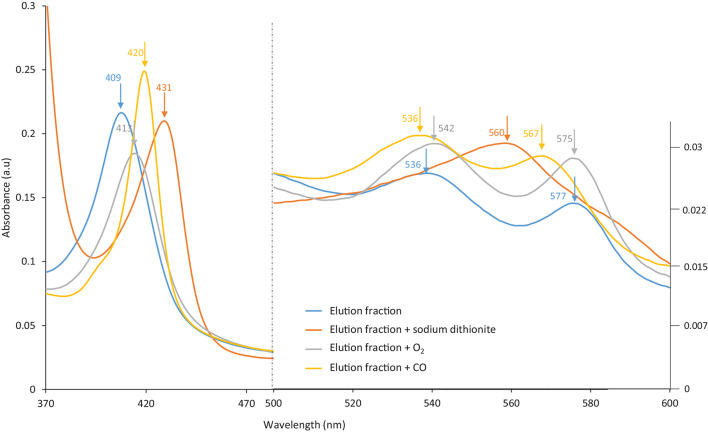
The spectra of the eluted HbF solution from MIP Am-CG column before and after treatment with Dithionite and CO gas.

Hb proteins can easily be oxidized during handling, and precautions must be taken to avoid such side reactions. Several methods are available to control oxidation. For instance, the HbF sample (pink-reddish color) was purged with carbon monoxide (CO) gas prior to purification in order to form HbF-CO, which stabilizes the heme group. It was noticed that the eluted protein solution from MIP Am-CG had changed into a slightly browner color. This observation indicates the transformation of the heme group into another oxidative state, particularly the ferric form (Ratanasopa et al., 2015; Jensen and Fago, 2018). The color changes in the elution solution were examined spectrophotometrically. Figure 7 shows that the absorbance peak of the eluted protein has shifted from 419 to 407 nm, and that the two peaks related to HbF-CO at 539 and 569 nm have disappeared. Based on the absorbance scan, the elution spectrum is better matched to hemichrome HbF. This phenomenon can be avoided by degassing the buffer solutions and using an extra oxygen scavenger such as ascorbic acid (Niki, 1991) to protect the heme group during the purification process (Smith and Nunn, 1984). The availability of the ligand-binding iron atom in the heme group in the fractions was assessed by adding sodium dithionite and then exposing the samples to oxygen and carbon monoxide gases. This transition from HbF hemichrome state to HbF-O_2_ and HbF-CO indicates that the changes in the heme groups are reversible for the “elution” fraction. Thus, the heme groups are still accessible and functional after the purification in the captured and eluted fraction (Figure 7).

The two possible sources that may cause the modification of the HbF in the “flow-through” fraction into irreversible hemichrome are the cryogel column (residual monomers after the polymerization) or the regeneration solution (acetic acid containing SDS). Control experiments were designed to evaluate the impact of each of these alternatives. Two small pieces of dried gel (from each column, NIP and MIP, 1 mg) was added to 1 mL of HbF-CO solution separately and stirred at room temperature for 1 h. The supernatant of each sample was scanned using a spectrophotometer, and HbF protein peaks were analyzed before and after incubation with cryogels (Supplementary Figure 5). The spectra in Supplementary Figure 5 suggest that the polymeric network did not influence the HbF solution to form hemichrome after this incubation. The HbF spectra show that the heme group is active and can react with oxygen and carbon monoxide after incubation. Hence, the polymeric network of the cryogelin itself had nodetrimental effect on the heme group changes during the purification process. The pieces of the NIP and MIP Am-CGs were then washed for 5 min with 1 mL of regeneration solution on a rocking table. The gels were subsequently washed with 20 mL of water and 2 × 4 mL of 0.1 M phosphate buffer with a pH of 6.0, for a minimum of 2 h. The washing solutions were changed several times to ensure the removal of SDS from the network. The pH of the supernatant was monitored between each wash.

Supplementary Figure 6 shows the changes in the HbF spectra before and after incubation with regenerated cryogels. From the spectra in Supplementary Figure 6, it is clear that the modification has occurred on the heme groups and that they have formed hemichrome. Although the small pieces of both cryogels (NIP and MIP) were washed excessively, the SDS was not entirely removed from their system. SDS is a small molecule that can diffuse inside the walls of the matrix and is therefore difficult to remove. On the other hand, this surfactant is a well-known reagent used to form hemichrome (Liu et al., 2007). Supplementary Figure 7 displays the spectra of HbF in different states. The spectra in Supplementary Figure 7A shows a very similar transition of the heme group in the elution fraction (Figure 7). This experiment has clarified that using an acetic acid solution containing SDS as a regeneration solution has a dramatic impact on the heme groups and can cause irreversible changes inside the protein molecule. During the control experiment with regenerated cryogels, it was noticed that the residual concentration of SDS in the MIP Am-CG was higher than that in the NIP Am-CG due to the appearance of a thin layer of foam inside the tube. Thus, the spectra in Supplementary Figure 7B shows the irreversible transformation of the heme group to its hemichrome form. This indicates that a more thorough washing step is required to remove the SDS. Thus, a more extensive washing step should be designed for the main separation column for removing the SDS. An alternative would be to replace the regeneration solution with another reagent, such as an oxalic acid solution.

## Conclusion

Fast separation of HbA from a non-clarified cell homogenate was carried by a large volume of composite cryogel in a chromatographic column has been reported for the first time in this work. This study demonstrated the possibility to construct molecularly imprinted composite cryogels on a large scale in the presence of the target molecule. The high selectivity of the matrix toward the template was confirmed in the presence of other proteins, impurities, and cell debrides. In addition, the ability of the imprinted cryogel to distinguish between the imprinted targets, HbA, vs. the HbF type was confirmed the selectivity of the recognition sites with high precision. The increase in the purification percentage in one-step treatment with high recovery demonstrates the advantage of using large cryogel columns in downstream processing compared to centrifugation and filtration. In addition, this work has illustrated that the suggested system can be reused continuously after a mild regeneration step. The cryogel column can be a proper replacement for these high energy and time-consuming methods. The elution fraction of the column can be directly guided to the other chromatography step-ups for fine polishing. The simplicity and reproducibility of the proposed technique can be applied to other types of cryogel columns for bioseparation purposes.

## Data Availability Statement

The raw data supporting the conclusions of this article will be made available by the authors, without undue reservation.

## Author Contributions

SH and KK designed and performed the experiments and drafted the manuscript. All authors revised the manuscript and have given approval to the final version of the manuscript.

## Conflict of Interest

The authors declare that the research was conducted in the absence of any commercial or financial relationships that could be construed as a potential conflict of interest.

## Publisher’s Note

All claims expressed in this article are solely those of the authors and do not necessarily represent those of their affiliated organizations, or those of the publisher, the editors and the reviewers. Any product that may be evaluated in this article, or claim that may be made by its manufacturer, is not guaranteed or endorsed by the publisher.
