# Supplementary material for: Rapid Separation of Human Hemoglobin on a Large Scale From Non-clarified Bacterial Cell Homogenates Using Molecularly Imprinted Composite Cryogels
Source: Front Bioeng Biotechnol. 2021 Oct 1;9:671229. doi: 10.3389/fbioe.2021.671229 (PMC8517190; doi:10.3389/fbioe.2021.671229)
Supplement: Supplementary file 1 [file Data_Sheet_1.docx]

**Supplementary information**

**Rapid separation of human hemoglobin on a large scale from non-clarified bacterial cell homogenates using molecularly imprinted composite cryogels**

Solmaz Hajizadeh^*^, Karin Kettisen, Leif Bülow, Lei Ye

Division of Pure and Applied Biochemistry, Department of Chemistry, Lund University, 22100 Lund, Sweden.

^*^Corresponding author: E-mail: [Solmaz.hajizadeh@tbiokem.lth.se](mailto:Solmaz.hajizadeh@tbiokem.lth.se), Telephone: +46 (0) 46 222 14 88

Figure S1. FTIR spectra of NIP and MIP Am-CG

Figure S2. Digital images of separation of HbA from a protein solution (BSA and HbA) from a 50 mL NIP Am-CG column. a) before loading the protein solution; b) during the loading step; c) after washing with phosphate buffer (0.1 M, pH 6); d) after elution with carbonate buffer (0.1 M, pH 9); e) top of the column from image c.

Figure S3. Digital images of NIP Am-CG column (150 mL) at different stages of HbA separation from a protein solution (BSA and HbA). A) before loading the protein solution; B) during the loading step; C) after washing with phosphate buffer (0.1 M, pH 6); D) after elution with carbonate buffer (0.1 M, pH 9).

Figure S4. Digital image of SDS-PAGE analysis of HbF from non-clarified cell homogenate. 1) Protein marker; 2) loaded fraction; 3) Flow-through fraction; 5) washing fraction; 6) Elution fraction.

Figure S5. The spectra of HbF solution before and after incubation with A) NIP Am-CG and B) MIP Am-CG.

Figure S6. The spectra of HbF solution before and after incubation with regenerated A) NIP Am-CG and B) MIP Am-CG.

Figure S7. The spectra of the heme group in A) HbF and B) HbF hemichrome at different states.
